# *Drosicha corpulenta* (Hemiptera: Monophlebidae) in an Arid New City: Phenology, Host Suitability, and Spatial Distribution of Overwintering Eggs

**DOI:** 10.3390/insects17010127

**Published:** 2026-01-22

**Authors:** Abdushalam Axpanmu, Wenhui Li, Changyue Liu, Zihan Yang, Xingyu Pu, Qizhi Liu, Shaoshan Wang

**Affiliations:** 1 Key Laboratory of Oasis Agricultural Pest Management and Plant Protection Resources Utilization, College of Agriculture, Shihezi University, Shihezi 832061, China; hasibaa@163.com (A.A.); 17799379914@163.com (Z.Y.); pusasa666@163.com (X.P.); lqzzyx126@126.com (Q.L.); 2 Xinjiang Production and Construction Corps Fourth Division Forestry and Grassland Service Center, Cocodala 835213, China; huihui820612@sohu.com (W.L.); lcyfang@163.com (C.L.); 3 Laboratory of Entomology and Nematology (LEN), College of Plant Protection, China Agricultural University, Beijing 100193, China

**Keywords:** *Drosicha corpulenta*, occurrence dynamics, host preference, overwintering eggs, spatial distribution

## Abstract

*Drosicha corpulenta* (Hemiptera: Monophlebidae) is a major polyphagous pest affecting street and garden trees in arid regions of northern China, causing increasing damage in newly developed cities like Cocodala, Xinjiang. This study was conducted from 2024 to 2025 to investigate this pest’s life cycle, key damage periods, and spatial distribution in seven host plants, focusing on nymph emergence, female soil entry, and overwintering egg distribution. The results show that *D. corpulenta* has one generation per year, which overwinters as eggs. Nymphs emerge in early March, and male pupation occurs from mid-April to early May. Females mate after the third molt in early to mid-May and enter the soil to lay eggs from late May to early June, with consistent timing over two years. The suitability of the host varied significantly: *Platanus × hispanica* was the most preferred, with the highest daily nymph emergence of 840.8 individuals in 2024 and 1196.0 in 2025, followed by *Prunus padus* and five other plant species (*Populus* spp., *Fraxinus chinensis*, *Styphnolobium japonicum*, *Pinus* spp., and *Malus spectabilis*). Female soil entry reached a maximum on 23 May (979.8 individuals^−1^ day^−1^) and gradually decreased with increasing distance from the trunk. Overwintering eggs showed no obvious azimuthal bias, but were mainly concentrated near the trunk (0–30 cm) and in shallow soil (0–10 cm; 179.8 eggs per 100 g composite soil sample per sampling point), decreasing sharply in number with distance and depth. Both Taylor’s power law and Iwao’s regression confirmed the aggregated distribution. This study identifies key phenological stages, highly susceptible hosts, and the near-trunk shallow soil layer as critical for oviposition and overwintering and provides a basis for precise monitoring and targeted control in urban green spaces.

## Simple Summary

*Drosicha corpulenta* is a major pest in urban gardens in China. This study was conducted from 2024 to 2025 in Cocodala City, Fourth Division of the Xinjiang Production and Construction Corps, to investigate the life cycle, host plant suitability, and spatial distribution of the species’ overwintering eggs. This pest has one generation per year and overwinters as eggs in soil. Nymphs emerge and climb trees in early March, and peak egg laying by females occurs in late May. Among seven common tree species, *Platanus acerifolia* and *Prunus padus* have been identified as the most suitable hosts, whereas *Sophora japonica*, *Pinus* spp., and *Malus spectabilis* are regarded as less suitable. Overwintering eggs are clustered in shallow soil within 30 cm of a trunk and at depths of less than 10 cm. These findings confirm and extend earlier work by Cui Meixiang et al. in temperate regions. Critical control periods include the nymph eclosion in early spring and adult egg laying; soil pesticides should be applied near tree bases to eliminate eggs. This study provides a scientific basis for the precise monitoring and targeted control of *Drosicha corpulenta* in urban greening.

## 1. Introduction

The quality of human settlements and biodiversity conservation is directly linked to the health and stability of urban green space ecosystems [1]. Habitat fragmentation and the simplification of host plant assemblages driven by urbanization create favorable conditions for outbreaks of phytophagous pests [2,3,4,5]. In recent years, studies have shown that urban warming and changes in vegetation structure can significantly affect the abundance, spatial distribution, and phenology of tree pests and their natural enemies, thereby compromising the health of street trees and the ecosystem services provided by urban forests [4,6,7,8,9,10,11,12]. Against this background, scale insects (including mealybugs and armored scales) have become a focal group in research on urban tree pests because they tend to form high-density aggregations on trunks, branches, and foliage [11,13].

*D. corpulenta* is an important polyphagous pest in urban greening systems in northern China and other arid regions of the country [14]. Nymphs and adult females feed on sap from the young branches and buds of host plants, leading to reduced tree growth, leaf yellowing, and premature defoliation. In severe cases, infestations can cause branch dieback and even the death of entire trees [15]. Concurrently, the copious excretion of honeydew promotes the growth of sooty mold, which degrades the esthetic quality of green spaces [16]. This increases the operational costs of urban forestry and landscape maintenance. To effectively manage the damage inflicted by *D. corpulenta*, Chinese researchers have conducted decades of foundational research and achieved substantial progress. Most existing studies have focused on temperate and semi-humid regions, such as the Guanzhong Plain in Shaanxi Province, and systematically elucidated the life cycle and phenology of this univoltine pest species. Key phenological windows have been clearly identified, including the hatching of overwintering eggs, nymph emergence and their ascent onto host trees, and the descent of adult females for oviposition. These findings provide precise phenological benchmarks for timing physical control measures, such as the application of trunk barriers, in early spring [17].

Previous research has consistently indicated that the overwintering egg sacs of *D. corpulenta* display an aggregated distribution within the soil, predominantly concentrated in loose topsoil in the immediate vicinity of a tree trunk [18]. Within integrated pest management frameworks, research has highlighted the importance of natural enemies, particularly *Rodolia limbata*, and developed predator–prey dynamic models to enhance and optimize control strategies [19,20]. From the perspective of urban insect ecology, habitat structure at both local and landscape scales, including vegetation complexity and the extent of impervious surfaces, significantly affects the abundance and spatial distribution patterns of pests and their natural enemies [2,3,21,22,23].

Host plants constitute the fundamental resource for insect survival and reproduction. Variation in host species, nutritional constituents, and volatile profiles can influence pest growth and development and, consequently, population dynamics [24,25]. Different host plants demonstrate notable variations in their suitability for and resistance to *D. corpulenta* (the cottony maple scale). However, which urban greening tree species in arid regions are highly susceptible hosts and which exhibit potential resistance to this pest remains uncertain. In addition, whether the life cycle timing of *D. corpulenta* is shaped by arid climatic conditions remains to be determined, and the timings of its key developmental stages must also be precisely delineated and monitored, including overwintering egg hatching, nymphal tree colonization, and adult mating and oviposition. Moreover, the spatial preferences of adult female *D. corpulenta* for oviposition in the soil and the distribution pattern of overwintering eggs directly influence the initial population density and occurrence range in the following year, forming a crucial basis for developing targeted management strategies for *D. corpulenta* in the future. To date, information remains scarce on the spatial ecology of *D. corpulenta* eggs [26,27,28]. The response mechanism of *D. corpulenta* to various environmental factors (i.e., soil temperature and soil moisture) remains unclear, which limits our comprehensive understanding of its population diffusion patterns.

Building on these knowledge gaps and research imperatives, this study focuses on Cocodala City in Xinjiang, a representative arid urban area experiencing increasingly severe infestations of *D. corpulenta*. Through systematic field investigations conducted over two consecutive years, this study aimed to precisely characterize the phenology of *D. corpulenta* in arid urban environments, evaluate differences in population dynamics across seven major host plant species, and elucidate the soil colonization behavior of adult females, as well as the vertical and horizontal spatial distribution patterns of their overwintering eggs. The findings of this study will deepen our understanding of the ecological adaptation mechanisms of *D. corpulenta* in extreme arid habitats, while also providing urban green space management departments in arid regions with scientifically grounded and operational guidance for localized, phenologically informed, and spatially targeted monitoring, as well as early warning and integrated pest management. Overall, this study holds substantial theoretical significance and practical relevance.

## 2. Materials and Methods

### 2.1. Experimental Materials

This study was conducted in the urban green belts of Cocodala City in the Xinjiang Uygur Autonomous Region (80°57′ E, 43°55′ N) during 2024–2025. The surveyed urban tree species were *Platanus × hispanica* (London plane tree), *Prunus padus* (bird cherry), *Populus* spp. (poplar trees), *Fraxinus chinensis* (Chinese ash), *Styphnolobium japonicum* (Japanese pagoda tree), *Pinus* spp. (pine trees), and *Malus spectabilis* (Chinese flowering crabapple); they were selected as the host trees in this study. These tree species were selected for their representativeness and prevalence in the study area, confirmed *D. corpulenta* infestation in preliminary surveys, relatively consistent management conditions, good tree health, and suitability for repeated observations.

The colored and yellow sticky traps used in the experiment were obtained from Linong Agricultural Science Co., Ltd (Lijiang, China). The colored traps comprised seven colors: yellow, white, red, blue, green, purple, and black. They had uniform dimensions of 25 cm × 20 cm with adhesive coating on both sides. Transparent plastic tape (20 cm wide) was procured from local markets and used to block the upward colonization of nymphs on tree trunks.

### 2.2. Field Investigation on the Life History of D. corpulenta

Field investigations into the life history of *D. corpulenta* were conducted from February to July in 2024 and 2025. At the onset of newly hatched nymphs emerging from the soil, 15 *Platanus × hispanica* trees with an average trunk circumference of approximately 70 cm (measured at 1.5 m above ground level) were selected in Cocodala City, Xinjiang Uygur Autonomous Region, and each individual was permanently tagged for identification. On each sample tree, one upper-canopy branch per cardinal direction (east, south, west, north) was selected and secured for observation, and a 60-mesh fine gauze cage was installed along each designated branch to prevent both inward and outward movement of *D. corpulenta* and exclude interference from natural enemies. After counting the insects, the cages were returned to the same branch. Beginning with soil emergence and the upward colonization of newly hatched nymphs, the developmental status of individuals within the gauze cages was monitored daily in 24 h intervals. Observations were conducted at 16:00 (4:00 p.m.) each day, and 20 individuals were examined per observation. Key life history events were recorded, including upward tree movement, molting, progression through nymphal instars, adult emergence, mating, and the downward migration of adult females into the soil for oviposition. Nymphal instars were determined primarily by the timing and presence of exuviae, and these determinations corroborated with observations of integument morphology and body length variation under a stereomicroscope.

### 2.3. Colonization of Different Host Trees by Nymphs

From mid-March to mid-April 2024 and from late February to mid-April 2025, surveys were performed to investigate soil emergence and upward colonization by newly hatched *D. corpulenta* nymphs on seven different host plant species. The chosen host species included *Platanus × hispanica*, *Prunus padus*, *Styphnolobium japonicum*, *Populus* spp., *Fraxinus chinensis*, *Pinus* spp., and *Malus spectabilis*. To ensure a randomized assessment, 15 individuals of each host species were selected and arranged in a randomized block design, consisting of three replicates with five trees per replicate. Under each tree, a soil thermometer was employed to measure the soil temperature at a vertical depth of 5 cm. The three blocks were defined by road segment and randomly selected from the study area; within each block, trees were chosen from the same green belt under consistent management conditions. To monitor nymph activity, a 20 cm wide transparent sticky barrier tape was positioned around the trunk of each sample tree at a height of 150 cm above the ground. Nymphs that adhered to the lower edge of the tape indicated the intensity of daily soil emergence and colonization. Throughout the colonization phase, researchers conducted daily counts of newly hatched nymphs within a 20 cm arc segment at the lower edge of the tape at a consistent time (16:00). This allowed for the identification of peak emergence dates and the duration of colonization for each host species in the study area.

### 2.4. Soil Colonization by Fecund Females

From 17 May to 10 June 2025, fifteen *Platanus × hispanica* trees with a trunk circumference of approximately 60 cm (measured at fifteen m above ground level) were selected within the urban green belts of Cocodala City, Xinjiang Uygur Autonomous Region. To capture adult females of *D. corpulenta* migrating downward to the soil for oviposition, yellow sticky traps (20 cm × 25 cm) were deployed at 3 horizontal distance gradients (0–30 cm, 30–60 cm, and 60–90 cm from the trunk base) along eight cardinal and intercardinal directions (E, W, S, N, NE, NW, SE, and SW, 45° apart), resulting in 24 traps per tree (three distance gradients × eight directions). A randomized block design was implemented with three replicates, each comprising five trees. The number of adult females adhered to each sticky trap was recorded every three days. Furthermore, the temperature of the day was recorded, and traps were replaced every three days to maintain consistent trapping efficiency for subsequent statistical analyses.

### 2.5. Spatial Distribution Survey of D. corpulenta Eggs

In late November 2024, 30 *Platanus × hispanica* trees with trunk circumferences of approximately 60 cm (measured at 1.5 m above ground level) were selected within the urban green belts of Cocodala City, Xinjiang Uygur Autonomous Region. A plot-based sampling design was implemented, in which three areas (plots) were randomly selected within the green belts under comparable management conditions, and ten healthy trees with a similar trunk size were sampled within each plot (30 trees in total). The use of ten trees per plot was intended to improve within-plot representativeness and reduce random variation among individual trees. The soil was sampled for each tree across two spatial dimensions: horizontal distance gradients (0–30 cm, 30–60 cm, and 60–90 cm from the trunk base, measured in four cardinal directions: east, west, south, and north) and a vertical depth gradient (0–5, 5–10, 10–15, and 15–20 cm below the soil surface). This design resulted in a total of 48 sampling points per tree (3 distances × 4 directions × 4 depths). At each sampling point, the soil samples were meticulously sieved in the field to eliminate large debris and subsequently spread uniformly on clean trays. Four subsamples were randomly selected from each sampling point and amalgamated into one composite sample. After thoroughly homogenizing the composite sample, 100 g of it was weighed using an electronic balance for egg enumeration. Nevertheless, the total number of sampling points remained unaltered. The number of eggs of *D. corpulenta* in each 100 g wet weight sample was tallied and documented for subsequent analysis. The egg counts for each tree at each sampling point were recorded without aggregating them into a single total at the plot level.

#### Analysis of Spatial Distribution Pattern and Aggregation Indices of *D. corpulenta* Overwintering Eggs

Each Platanus tree (*n* = 30) was treated as an independent sample unit. For each tree, the egg counts from all subsamples (i.e., all combinations of cardinal direction × horizontal distance × soil depth) were used to calculate the mean (*m*) and variance (*S*^2^). The dispersion coefficient of David and Moore (Bell’s coefficient) was computed as follows [28,29]:

(1) $$C=\frac{S^{2}}{m},$$

Here, *C* = 1, *C* > 1, and *C* < 1 represent random, aggregated, and uniform distributions, respectively. David and Moore’s clumping index was calculated as follows [28,29]:

(2) $$I\;=\frac{S^{2}}{m\;-1}$$

Here, *I* = 0, *I* > 0, and *I* < 0 indicate a random, distributed, and uniform distribution, respectively. The Morisita dispersion index was calculated as follows [29,30]:

(3) $$I_{\delta }\;=\frac{n\sum \left(fx^{2}-N\right)}{N\;(N\;-1)}$$

where *N* is the total egg count, and *n* is the number of samples, and it can be approximated as follows [29,30]:

(4) $$I_{\delta }=\frac{m*}{m\;-1/n}$$

Here, $I_{\delta }$ = 1, $I_{\delta }$ > 1, and $I_{\delta }$ < 1 indicate random, aggregated, and uniform distributions, respectively. The aggregation parameter (*K*) of the negative binomial distribution was computed as follows [29,30,31,32]:

(5) $$K=\frac{m^{2}}{S^{2}-m}$$

Smaller *K* values indicate stronger aggregation, while *K* values greater than 8 suggest a distribution closer to a Poisson random pattern. Lloyd’s mean crowding was calculated as [33,34]

(6) $$m*=\frac{m+S^{2}}{m-1}$$

In addition, the ratios of m*/*m* = 1, >1, and <1 correspond to random, aggregated, and uniform distributions, respectively. The Cassie–Kuno aggregation index was defined as follows [29,35]:

(7) $$CA=\frac{S^{2}-m}{m^{2}}$$

Here, CA=0 , CA > 0, and CA < 0 indicate a random, aggregated, and uniform distribution, respectively. Based on regression analysis, Iwao’s m*–*m* model was expressed as [29,36]

(8) m∗=α+βm

Here, α represents the mean crowding of the basic component, and β indicates its spatial distribution pattern. When α = 0, the basic component consists of single individuals; α > 0 indicates clusters of individuals, and α < 0 indicates repulsion among individuals. β = 1, β > 1, and  β < 1 correspond to random, aggregated, and uniform distributions, respectively. Taylor’s power law describes the log–log relationship between variance and mean as [29,32]

(9) $$\log_{10}S^{2}=\log_{10}a+b\;\log_{10}m$$

When $\log_{10}a$ = 0 and *b* = 1, the distribution is random; when $\log_{10}a$ > 0 and *b* = 1, aggregation is density-independent; when $\log_{10}a$ > 0 and *b* > 1, aggregation is density-dependent; and when $\log_{10}a$ < 0 and *b* < 1, the distribution is uniform.

### 2.6. Measurement and Data Analysis

All data were organized in Excel, and statistical analyses were carried out using SPSS 26.0 software. Differences in daily nymph emergence and total annual occurrence among host tree species were evaluated through one-way analysis of variance (ANOVA), accompanied by Tukey’s post hoc test to determine the significance of the differences. Statistical significance was set at *p* < 0.05 for all analyses. Egg count and soil entry data were modeled utilizing the glmmTMB function from the glmmTMB package [37]. Considering the overdispersion and zero inflation present in the egg count and soil entry data, a negative binomial distribution (link = “log”) was selected because it can effectively handle overdispersion. To deal with the zero-inflation component, formula = ~direction + distance was employed for modeling. The fixed effects within the model encompassed direction and distance (vertical depth), whereas the random effects accounted for the nested structure of regions (subplots) and tree groups, as well as the variations across dates. Variance analysis for egg count and soil entry was performed using the Anova function from the car package [38]. Multiple comparisons for egg count and soil entry were conducted with the use of the emmeans package [39], and Tukey’s method was applied for *p*-value adjustment. The aggregation index for overwintering eggs, along with Iwao regression and Taylor’s power law fitting, was computed according to the methods described in the relevant literature [29,30].

## 3. Results

### 3.1. Life History Survey of D. corpulenta in Cocodala City

*D. corpulenta* exhibited a univoltine life cycle in Cocodala City, as indicated in Table 1. The species overwintered at the egg stage, located within oothecae in the soil near tree bases. Egg hatching began in late February, with newly hatched nymphs remaining in the soil. In early March (14 March 2024; 2 March 2025), nymphs emerged from the soil and ascended the trees. Newly hatched nymph activity peaked around mid-March and concluded by early April. Initially, newly hatched nymphs clustered on buds, bud axils, and branch crotches, feeding on phloem by piercing and sucking. The developmental process exhibited sexual dimorphism: males underwent two molts and pupated within protective cottony shelters, with a pupal duration of approximately 10 days. In contrast, females lasted through three molts before reaching adulthood. Adult males emerged in May, mated high in the canopy, and typically died 3 to 5 days post-mating. Fertilized females continued to feed until early to mid-June, after which they transitioned to cool, moist microhabitats in the topsoil to construct oothecae. They laid eggs and subsequently died, completing their life cycle, as the eggs would then overwinter until the following spring.

### 3.2. Occurrence Dynamics Survey of Newly Hatched Nymphs of D. corpulenta on Different Host Plants

**Figure 1:**
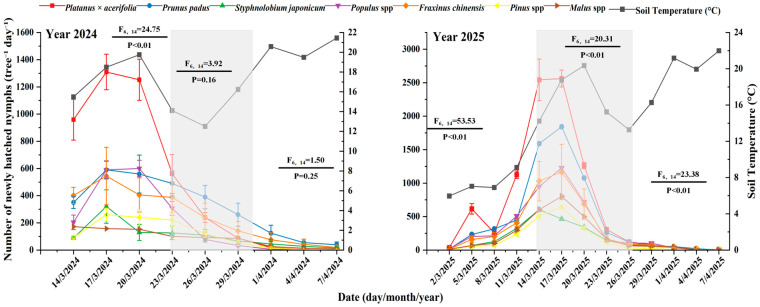
Investigation of the occurrence dynamics of first-instar nymphs of *D. corpulenta* on different host plants in 2024 and 2025 (*n* = 15). Note: In this study, nymph emergence was divided into three stages: early, middle, and late. The gray-shaded area in the figure represents the middle stage.

The emergence and colonization dynamics of newly hatched *D. corpulenta* nymphs were consistent across various host species (Figure 1). During the two-year soil temperature detection process, the soil temperatures during the peak periods were discovered to be relatively high. The initial emergence occurred in early March, with a peak mid-March and conclusion by early April, maintaining a similar duration across the seven host species (Figure 1). In 2024, during the early emergence phase (14–20 March), significant differences in daily emergence density were observed among hosts (F6,14 = 24.75, *p* < 0.01), but no notable inter-host differences were found during the middle (23–29 March; F6,14 = 3.92, *p* = 0.16) or late stages (1–7 April; F6,14 = 1.50, *p* = 0.25). Notably, the populations of *Malus spectabilis* and *Pinus* spp. remained low (Figure 1).

**Figure 2:**
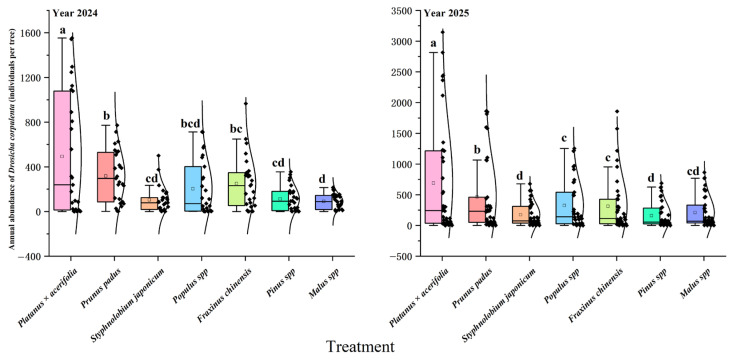
Annual total emergence of newly hatched nymphs of *D. corpulenta* on different host plants in 2024 and 2025. Note: Boxplots in different colors represent different host plants. The box indicates the interquartile range (Q1–Q3), the line inside the box shows the median, and the whiskers indicate the data range. Black dots represent observations from each replicate sample; the outer black curves show the density (distribution shape) of the data. Different lowercase letters indicate significant differences among treatments within the same year (*p* < 0.05), whereas the same letter indicates no significant difference.

In 2025, the trends followed those of 2024 but with higher nymph densities. During the early (14–21 March) emergence, there were significant differences among hosts (F6,14 = 53.53, *p* < 0.01), and these differences continued throughout the middle (14–26 March; F6,14 = 20.31, *p* < 0.01) and late stages (26 March–7 April; F6,14 = 23.38, *p* < 0.005). The peak daily soil emergence reached 1772.0 nymphs tree^−1^ day^−1^ individuals on 19 March 2024, and rose to 3276.4 nymphs tree^−1^ day^−1^ individuals on 17 March 2025, indicating a substantial increase in population density in 2025 (Figure 1). The ranking of host suitability remained stable, with *Platanus × hispanica* identified as the most susceptible host, with a peak daily emergence of 840.8 nymphs tree^−1^ day^−1^ individuals per day on 18 March 2024, and 1196.0 nymphs tree^−1^ day^−1^ individuals per day on 16 March 2025, followed by *Prunus padus*, with *Populus* spp. and *Fraxinus* spp. (Figure 1). At intermediate levels, *Styphnolobium japonicum*, *Pinus* spp., and *Malus spectabilis* exhibited consistently low densities. Additionally, comparisons of annual totals confirmed that the mean annual density on *Platanus × hispanica* and *P. padus* was significantly higher than that on other hosts (Figure 2), with peak and mean densities generally greater in 2025 than in 2024, particularly for *P. acerifolia* and *P. padus* (Figure 2).

### 3.3. Survey on the Soil Colonization Dynamics of Adult Female D. corpulenta

**Figure 3:**
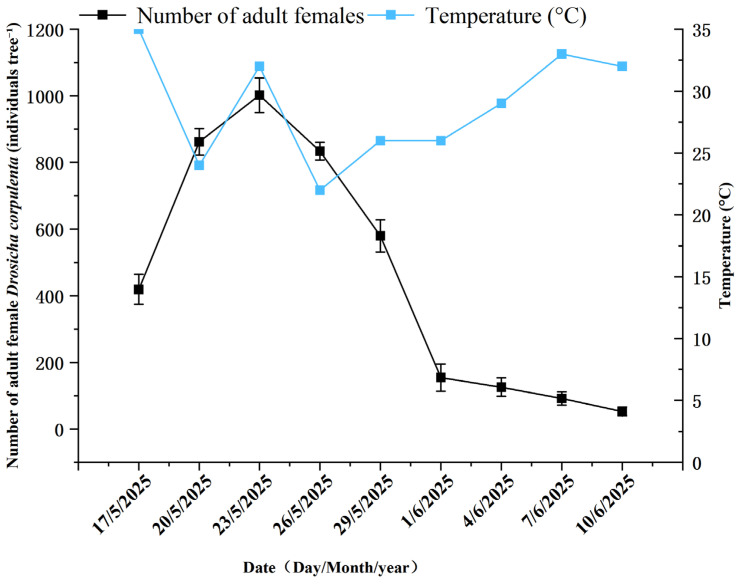
Occurrence dynamics of the number of *D. corpulenta* female adults entering the soil (*n* = 15).

**Figure 4:**
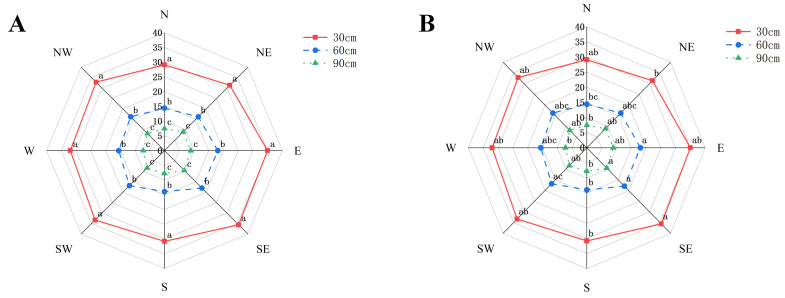
Radar charts of *D. corpulenta* adult female densities. (**A**) Different directions and distances from the tree trunk. (**B**) Different directions at the same distance. Note: Different letters indicate significant differences (*p* < 0.05), whereas the same letter indicates no significant difference.

The daily soil colonization density of adult *D. corpulenta* exhibited a pronounced temporal peak in mid-May (Figure 3). Furthermore, the temperatures typically remained at a relatively high level. From mid-May, adult females began descending from host trees; the density increased rapidly to 861.9 individuals day^−1^ on 20 May, and the temperature was registered at 24 °C. A maximum of 979.8 individuals day^−1^ was reached on 23 May. The temperature reached 32 °C and remained elevated with 834.1 individuals day^−1^ on 26 May. Thereafter, the density of adult females gradually declined and remained low from early to mid-June. Analysis of variance (Table 2) indicated a highly significant effect of horizontal distance from the trunk on female soil entry density (χ^2^ = 2532.67, *p* < 0.001), whereas the azimuth × distance interaction was not significant (χ^2^ = 12.13, *p* = 0.6). Spatially, colonization density decreased significantly with increasing horizontal distance from the trunk (Figure 4). Within 0–30 cm of the trunk, colonization density was highest and inter-azimuth differences were small; the southeast azimuth exhibited a marginally higher density (35.59 ind. day^−1^). At 30–60 cm, the overall density was significantly lower than that at 0–30 cm, with densities in the east and southeast azimuths significantly exceeding those in the west and northwest. At 60–90 cm, colonization density reached its minimum, and there were no significant azimuthal differences.

### 3.4. Survey on the Spatial Distribution of D. corpulenta Eggs

#### 3.4.1. Spatial Distribution of *D. corpulenta* Eggs Across Soil Depths, Azimuths, and Horizontal Distances from Tree Trunks

**Figure 5:**
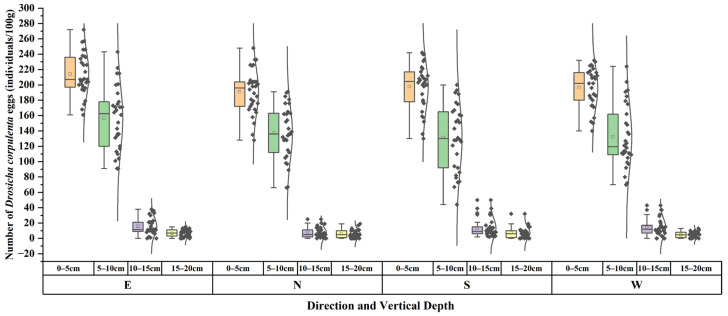
Vertical distribution of *D. corpulenta* eggs in different directions (*n* = 30). Note: Boxplots in different colors represent different vertical depths. The box indicates the interquartile range (Q1–Q3), the line inside the box shows the median, and the whiskers indicate the data range. Black dots represent observations from each replicate sample; the outer black curves show the density (distribution shape) of the data.

**Figure 6:**
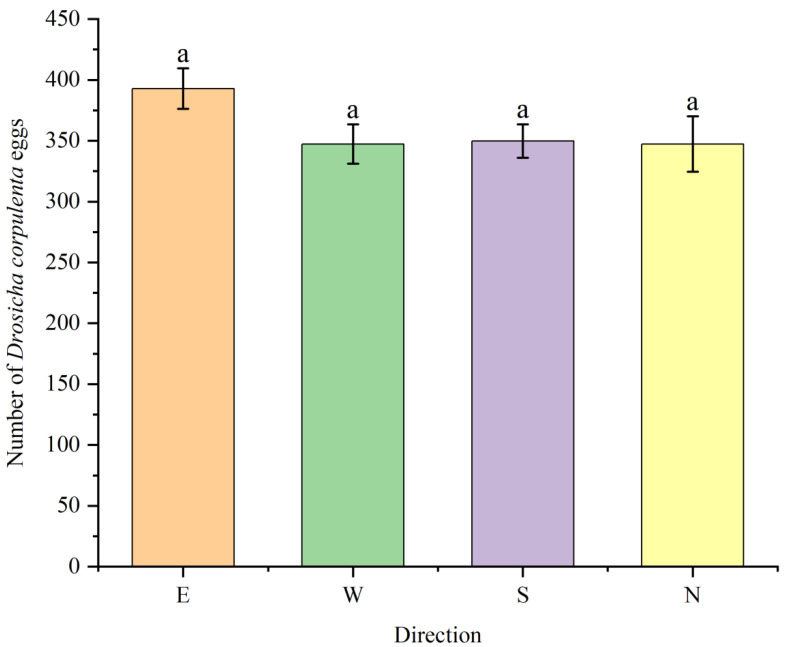
Radar chart of population density of *D. corpulenta* eggs at the same soil depth along different directions (*n* = 30). Note: Different letters indicate significant differences (*p* < 0.05), whereas the same letter indicates no significant difference.

This study found that the density of overwintering *D. corpulenta* eggs did not significantly differ among the four azimuth directions (east, south, west, north), suggesting that the azimuth has a minimal effect on their spatial distribution. In contrast, a significant variation in egg density was observed based on the soil vertical depth and horizontal distance from the tree trunks (Figure 5 and Figure 6). Specifically, the eggs were mainly found in the top 0–10 cm soil layer, with the highest density recorded in the 0–5 cm range, which was considerably greater than that in the 5–10 cm layer (F = 496.2, *p* < 0.01). The density in the 10–20 cm layer was notably lower, indicating a preference for shallow soil close to the surface. Horizontally, the egg density showed a significant decline with increasing distance from the tree trunk: 179.8 eggs per 100 g composite soil sample per sampling point within the 0–30 cm range, decreasing to 133.4 at 60 cm, and then further to 79.77 at 90 cm(Figure 6). However, there were no significant differences in egg density among the azimuths at either the 30 cm or 90 cm horizontal distance.

#### 3.4.2. Spatial Distribution Pattern of *D. corpulenta* Eggs

**Figure 7:**
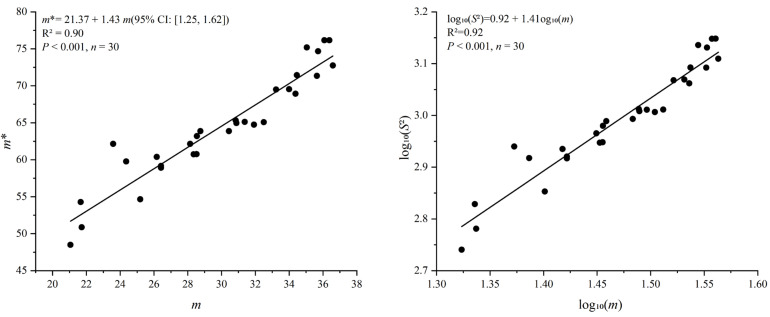
Iwao’s patchiness and Taylor’s power law regressions for overwintering eggs of *D. corpulenta* (*n* = 30).

Based on the aggregation indices, the overwintering eggs of *D. corpulenta* consistently exhibited an aggregated distribution, as indicated by *I* > 0, m*/*m* > 1, *CA* > 0, *C* > 1, and *K* > 0, confirming a clear aggregated spatial pattern in soil (Table 3). With Iwao’s m*–*m* regression, egg distribution was fitted as m* = 21.37 + 1.43 *m* (R^2^ = 0.91, *p* < 0.001, *n* = 30), with *β* = 1.43 (95% CI: 1.25–1.62; >1) and *α* = 21.37 (95% CI: 15.77–26.97; >0), suggesting clusters as the basic distribution unit and reinforcing the pronounced aggregation(Figure 7). Taylor’s power law further yielded log_10_*S*^2^ = 0.924 + 1.41·log_10_*m* (R^2^ = 0.925, *p* < 0.001, *n* = 30), with *b* = 1.41 (95% CI: 1.25–1.56; >1) and *a* = 0.924 (95% CI: 0.70–1.15), supporting aggregation, with aggregation intensity increasing as egg density increases (Figure 7).

## 4. Discussion

This study is the first to document that *D. corpulenta* completes one generation per year in the arid urban environment of Cocodala City in Xinjiang. Overwintering eggs were localized in the soil layer surrounding tree trunks, with key phenological time nodes differing markedly from those reported in humid regions [15,17]. In humid regions of eastern China, egg hatching typically begins in late January. In contrast, in Cocodala (Xinjiang), nymph emergence from the soil in our study occurred in early March, representing a delay of approximately 5–6 weeks compared with the humid regions in eastern China. This delay is likely attributable to slower spring warming and stronger soil thermal insulation under arid conditions [25]. Nymphal development followed patterns consistent with previous studies [40], with the shallow 0–10 cm soil layer providing a thermally stable microhabitat favorable for early egg hatching despite large diurnal temperature fluctuations. Ascent of nymphs begins in late February, coinciding with rising air temperatures yet preceding extreme heat, thereby minimizing low-temperature injury and enabling timely access to nutrients from sprouting shoots, reflecting a key ecological adaptation.

Males undergo two molts and pupate in late April (~10 days), with adult emergence and mating occurring from early to late May. Females undergo three molts and continue feeding on host branches until they descend for oviposition. Prolonged female feeding likely represents an adaptive response to arid-region stressors, including limited host nutrients and water availability, facilitating the accumulation of reserves for oogenesis and ensuring overwintering egg quantity and quality, thereby enhancing population survival [41]. Fertilized females oviposited under cool, moist soil clods or within soil surrounding tree trunks while secreting white cotton-like ovisacs, reducing egg desiccation and predation, and protecting against mechanical disturbances, which is a critical adaptive mechanism for population persistence [42]. This life history pattern involves one generation per year with overwintering eggs concentrated near the main trunk base; it aligns with observations of soft-scale insects (*Parthenolecanium* spp.) on urban street trees in the southeastern United States [43], demonstrating that urban tree pests can form highly adapted single-habitat populations under relatively stable tree species–microclimate combinations. However, the 60-mesh gauze cages used in this study may have altered within-cage microclimatic conditions and partially restricted the access of natural enemies, potentially affecting survival and population dynamics under natural conditions. Future studies should include uncaged controls and concurrent monitoring of microclimate inside and outside cages to quantify cage effects and improve the reliability of our conclusions.

Host plant suitability is a key determinant of *D. corpulenta* population density and outbreak potential [25,44]. Systematic surveys over two consecutive years confirmed that *Platanus × hispanica* (French plane tree) and *Prunus padus* (bird cherry) are more suitable hosts, whereas poplars (*Populus* spp.) and ashes (*Fraxinus* spp.) are moderately suitable hosts, and *Styphnolobium japonicum* (Chinese scholar tree), pines (*Pinus* spp.), and crabapples (*Malus* spp.) are less suitable hosts. This host suitability pattern exhibited temporal stability in the urban green spaces of arid regions. As a dominant urban street tree, *Platanus × hispanica* possesses smooth bark, abundant sap in tender branches, and balanced nutrient composition. Its buds sprout early and abundantly in spring, providing sufficient nutritional resources for newly hatched nymphs, making it the preferred host of *D. corpulenta* [45].

The phenology of *D. corpulenta* nymphs on different host plants followed an early spring unimodal pattern, although significant differences were observed in peak density and duration [25,41]. Surveys conducted in 2024 and 2025 consistently showed that the daily maximum emergence of nymphs on *Platanus × hispanica* was significantly higher than that on other hosts, with its total annual occurrence ranking first. In contrast, nymph abundance on *S*. *japonica*, *Pinus* spp., and *Malus* spp. remained low, likely due to intrinsic resistance traits: *S. japonica* branches are highly lignified with thick bark, impeding nymphal piercing and sucking, while *Malus* spp. may reduce sap nutrient content and accelerate branch lignification to resist feeding. Overall, nymph densities across all hosts were higher in 2025 than in 2024, with the increase being most pronounced on highly susceptible hosts. These patterns indicate that under favorable environmental conditions, highly susceptible hosts provide abundant resources that facilitate rapid population growth, whereas insect-resistant hosts limit population expansion. Accordingly, optimizing host plant composition in urban greening by increasing the proportion of insect-resistant species such as *S. japonica* and *Malus* spp. and reducing the continuous planting of *Platanus × hispanica* and *p. padus* can mitigate *D. corpulenta* outbreak risk at the source. Between 2024 and 2025, the population of *D. corpulenta* significantly increased. Possible contributing factors include inter-annual climate variation, the species’ high reproductive capacity and overwintering success, leading to cumulative population effects, and differences in management practices. Future studies will focus on systematically recording environmental and management data to better understand the factors driving population fluctuations.

The spatial pattern of soil entry and oviposition by female *D. corpulenta* directly determines the distribution of overwintering eggs, thereby affecting the density of nymphs ascending host trees and the potential damage range in the subsequent year [46]. Female soil entry peaked in mid-May, which coincided with the local warming period and seasonal soil moisture conditions. Horizontally, the number of soil-entering females decreased significantly with increasing distance from the trunk, with over-concentration within 0–30 cm. This pattern corresponds to the biological tendency of female *D. corpulenta* to select oviposition sites near host plants [46,47]. The soil surrounding tree trunks, shaded by the canopy, may maintain relatively higher humidity and reduced temperature fluctuations, potentially providing a more stable microenvironment for egg overwintering.

Direction had no significant effect on female soil entry, whereas female abundance decreased significantly with increasing distance from the trunk, indicating that horizontal distance is the primary determinant of the distribution of soil entry/oviposition site. The results show no significant interaction between direction and distance, further confirming that horizontal distance is the dominant factor governing female soil entry. These findings support the precise management of *D. corpulenta*, suggesting that targeted treatment of soil within 0–30 cm of tree trunks during peak female soil entry and oviposition can effectively reduce the overwintering egg bank.

Spatial analysis of *D. corpulenta* overwintering eggs revealed a pronounced aggregated distribution in the soil, with aggregation intensity increasing alongside population density [29,48]. Both aggregation indices and regression analyses corroborated this pattern [27]. Egg aggregation primarily results from female oviposition behavior, as *D. corpulenta* females lay eggs in clusters in favorable microhabitats. Vertical and horizontal spatial factors, such as soil depth and distance from the trunk, strongly influence egg aggregation [26,49]. Vertically, eggs were concentrated in the 0–10 cm shallow soil layer, peaking at 0–5 cm, which may indicate that this layer has optimal soil temperature, humidity, and aeration conditions. Shallow soil could satisfy the thermal requirements for hatching while potentially avoiding hypoxia and low moisture in deeper layers. Horizontally, egg density declined with increasing distance from the trunk, mirroring the distribution of soil-inhabiting female adults.

Aggregated egg distribution confers ecological advantages, including enhanced thermal insulation, moisture retention, and protection against natural enemies, thereby promoting survival and reproductive success. These patterns conform to classical spatial ecology models, including Taylor’s power law and Iwao’s patchiness regression [29,38,50,51], and align with field-observed aggregations in pests such as *Phyllocnistis citrella*, *Phenacoccus solenopsis*, and *Pseudaulacaspis pentagona* [26,27,48,49]. The microenvironment near tree trunks in urban *Platanus × hispanica* forests in Cocodala City serves as a key site for *D. corpulenta* egg aggregation and should be prioritized for pest monitoring and control. Recommended management practices target the early-spring nymph ascent and the subsequent adult oviposition period, which are considered critical windows for controlling this pest. During the nymph ascent stage, physical measures such as tree-trunk barriers (e.g., sticky bands) and trunk-applied insecticide bands or rings can be used to limit upward movement and can be integrated with appropriate chemical and biological control options to suppress population spread. During the adult oviposition period, soil cultivation and soil-applied insecticide treatments have been suggested to reduce the number of adult females entering the soil for oviposition, as part of an integrated management strategy that may also incorporate chemical and biological measures [18,40].

## 5. Conclusions

This study systematically characterized the life history of *D. corpulenta* in the newly established arid urban area of Cocodala City, Xinjiang, demonstrating that the species completes one generation per year and overwinters as egg sacs in the soil surrounding tree trunks. Key phenological events, including overwintering egg hatching, nymphal tree ascent, female adult soil entry, and oviposition, were precisely documented. Significant interspecific variation in more suitable hosts was observed: *Platanus* × *hispanica* (London plane tree) and *Prunus padus* (bird cherry) were highly susceptible, whereas *S. japonica* (Chinese scholar tree), *Pinus* spp. (pines), and *Malus spectabilis* (Chinese crabapple) were determined to have low suitability. Female adults entering the soil and overwintering eggs exhibited a pronounced aggregated distribution in the soil surrounding trunks, with core aggregation zones concentrated within a 0–30 cm radial distance from trunks and 0–10 cm shallow soil layer. Based on these findings, we propose the following targeted management strategies for *D. corpulenta* in Cocodala City: prioritize intervention during early spring nymph ascent and peak female soil entry, implement targeted monitoring and soil-based control in near-trunk shallow layers, and optimize the proportion of highly susceptible host species in urban green space planning. These measures aim to manage *D. corpulenta* in a precise, cost-effective, and environmentally sustainable manner.

## Figures and Tables

**Table 1:** Phenology (life cycle) of *D. corpulenta* in Cocodala City during 2024–2025.

Developmental Stage	2024	2025	Remarks
Crawlers/newly hatched nymphs	Early March–early April	Early March–early April	First emergence from soil and dispersal onto host trees.
1st instar nymphs	Late March–early April	Late March–early April	Reached dominance shortly after crawler emergence.
2nd instar nymphs	Early April–mid-April	Early April–mid-April	Instar succession following the 1st instar.
Female 3rd instar nymphs → adult eclosion	Mid-/late April–early May	Mid-/late April–early May	Females completed the 3rd molt and eclosed to adults on trees.
Male pupae	Mid-/late April–early May	Mid-/late April–early May	Males began pupation while females molted to adults.
Adults: mating period	Early May–mid-/late May	Early May–mid-/late May	Adults present on trees; mating observed.
Females entering soil and egg sacs.	Late May–early June	Late May–early June	Females descended from trees, entered the soil, and formed egg sacs.
Eggs overwinter in egg sacs	Early June–early March of next year	Early June–early March of next year	Eggs overwintered inside egg sacs; hatching started in early March.

Note: “Early”, “mid(dle)”, and “late” indicate days 1–10, 11–20, and 21–end of the month, respectively. Phenological trends were identical in 2024 and 2025; therefore, the same periods are shown for both years.

**Table 2:** Generalized linear model (GLMM) results for the effects of direction and distance from the trunk base on the number of *D. corpulenta* adult females entering the soil.

Source of Variation	df	χ^2^	*p*
Direction	7	69.64	<0.001
Distance from trunk	2	2532.67	<0.001
Direction × distance	14	12.13	0.6

Note: *p*-values less than 0.001 are shown as <0.001.

**Table 3:** The figure shows the mean egg density (eggs/100 g) at different distances.

Tree ID	Mean	Standard Deviation	Variance *S*^2^	Clumping Parameter *I*	Cassie Index *CA*	Mean Crowding m*	Patchiness m*/*x*	Diffusion Coefficient	Negative Binomial *K*
1	35.71	36.77	1352.08	37.86	1.03	72.57	2.03	36.86	0.97
2	28.15	30.38	923.19	32.8	1.13	59.95	2.13	31.8	0.89
3	26.17	29.34	861.04	32.91	1.22	58.07	2.22	31.91	0.82
4	28.54	30.89	954.38	33.44	1.14	60.98	2.14	32.44	0.88
5	30.88	31.92	1018.62	32.99	1.04	62.87	2.04	31.99	0.97
6	34.38	33.96	1153.43	33.55	0.95	66.93	1.95	32.55	1.06
7	35.04	36.97	1366.76	39.0	1.08	73.05	2.08	38.0	0.92
8	36.08	37.5	1406.16	38.97	1.05	74.05	2.05	37.97	0.95
9	33.23	34.19	1169.29	35.19	1.03	67.42	2.03	34.19	0.97
10	28.52	29.79	887.32	31.11	1.06	58.63	2.06	30.11	0.95
11	26.42	28.85	832.16	31.5	1.15	56.92	2.15	30.5	0.87
12	34.46	35.18	1237.53	35.91	1.01	69.37	2.01	34.91	0.99
13	31.92	31.86	1015.18	31.81	0.97	62.72	1.97	30.81	1.04
14	31.38	32.02	1025.3	32.68	1.01	63.05	2.01	31.68	0.99
15	28.35	29.76	885.77	31.24	1.07	58.59	2.07	30.24	0.94
16	28.75	31.22	974.4	33.89	1.14	61.64	2.14	32.89	0.87
17	36.38	37.51	1407.13	38.68	1.04	74.06	2.04	37.68	0.97
18	26.42	28.74	826.12	31.27	1.15	56.69	2.15	30.27	0.87
19	32.5	32.04	1026.51	31.58	0.94	63.08	1.94	30.58	1.06
20	34.0	34.25	1172.81	34.49	0.99	67.49	1.99	33.49	1.02
21	30.44	31.37	984.12	32.33	1.03	61.77	2.03	31.33	0.97
22	24.35	28.76	826.91	33.95	1.35	57.31	2.35	32.95	0.74
23	36.58	35.87	1286.93	35.18	0.93	70.76	1.93	34.18	1.07
24	35.65	35.16	1236.57	34.69	0.95	69.34	1.95	33.69	1.06
25	21.67	25.96	674.1	31.11	1.39	51.78	2.39	30.11	0.72
26	23.58	29.51	870.93	36.93	1.52	59.51	2.52	35.93	0.66
27	21.06	23.46	550.19	26.12	1.19	46.18	2.19	25.12	0.84
28	30.83	32.07	1028.74	33.36	1.05	63.2	2.05	32.36	0.95
29	21.73	24.58	604.03	27.8	1.23	48.53	2.23	26.8	0.81
30	25.19	26.7	712.92	28.3	1.08	52.49	2.08	27.3	0.92

Note: This table presents the mean, standard deviation (SD), and variance of overwintering eggs per surveyed tree, along with various aggregation indices (*I*, *CA*, m*, m*/*m*, *C*, and *K*). The calculation methods for these aggregation indices are described in the Materials and Methods section.

## Data Availability

The original contributions presented in this study are included in the article. Further inquiries can be directed to the corresponding author.
